# The impact of health literacy and life style risk factors on health-related quality of life of Australian patients

**DOI:** 10.1186/s12955-016-0471-1

**Published:** 2016-05-04

**Authors:** Upali W. Jayasinghe, Mark Fort Harris, Sharon M. Parker, John Litt, Mieke van Driel, Danielle Mazza, Chris Del Mar, Jane Lloyd, Jane Smith, Nicholas Zwar, Richard Taylor, On behalf of the Preventive Evidence into Practice (PEP) Partnership Group

**Affiliations:** Centre for Primary Health Care and Equity, Level 3 AGSM, University of New South Wales Australia, Sydney, NSW 2052 Australia; Discipline of General Pratice, School of Medicine, Flinders University, Adelaide, South Australia Australia; Discipline of General Practice, University of Queensland, Brisbane, QLD Australia; Department of General Practice, Monash University, Notting Hill, VIC Australia; Faculty of Health Sciences and Medicine, Bond University, Gold Coast, QLD Australia; School of Public Health and Community Medicine, University of New South Wales Australia, Sydney, NSW Australia

**Keywords:** Quality of life, Health literacy, Life style risk factors, SF-12 version 2, Physical component score, Mental component score, Multilevel regression analysis

## Abstract

**Background:**

Limited evidence exists regarding the relationship between health literacy and health-related quality of life (HRQoL) in Australian patients from primary care. The objective of this study was to investigate the impact of health literacy on HRQoL in a large sample of patients without known vascular disease or diabetes and to examine whether the difference in HRQoL between low and high health literacy groups was clinically significant.

**Methods:**

This was a cross-sectional study of baseline data from a cluster randomised trial. The study included 739 patients from 30 general practices across four Australian states conducted in 2012 and 2013 using the standard Short Form Health Survey (SF-12) version 2. SF-12 physical component score (PCS-12) and mental component score (MCS-12) are derived using the standard US algorithm. Health literacy was measured using the Health Literacy Management Scale (HeLMS). Multilevel regression analysis (patients at level 1 and general practices at level 2) was applied to relate PCS-12 and MCS-12 to patient reported life style risk behaviours including health literacy and demographic factors.

**Results:**

Low health literacy patients were more likely to be smokers (12 % vs 6 %, *P* = 0.005), do insufficient physical activity (63 % vs 47 %, *P* < 0.001), be overweight (68 % vs 52 %, *P* < 0.001), and have lower physical health and lower mental health with large clinically significant effect sizes of 0.56 (B (regression coefficient) = −5.4, *P* < 0.001) and 0.78(B = -6.4, *P* < 0.001) respectively after adjustment for confounding factors. Patients with insufficient physical activity were likely to have a lower physical health score (effect size = 0.42, B = −3.1, *P* < 0.001) and lower mental health (effect size = 0.37, B = −2.6, *P* < 0.001). Being overweight tended to be related to a lower PCS-12 (effect size = 0.41, B = −1.8, *P* < 0.05). Less well-educated, unemployed and smoking patients with low health literacy reported worse physical health. Health literacy accounted for 45 and 70 % of the total between patient variance explained in PCS-12 and MCS-12 respectively.

**Conclusions:**

Addressing health literacy related barriers to preventive care may help reduce some of the disparities in HRQoL. Recognising and tailoring health related communication to those with low health literacy may improve health outcomes including HRQoL in general practice.

## Background

Health literacy is defined by the Institute of Medicine of the National Academies, USA as the degree to which individuals have the capacity to obtain, process and understand basic health information and services needed to make appropriate health decisions [1]. There are two ways of conceptualising health literacy: a risk factor or an asset. Health literacy as a risk factor fits best in clinical settings. It focuses on improved communication between doctors and patients [2]. Assets are a set of capabilities needed for everyday life in order to make decisions that affect ones’ health.

Low heath literacy is common in Australia. The 2006 Adult Literacy and Life Skills Survey (Australia) found 60 % of adults to be performing at the lowest levels of health literacy when assessed for prose literacy, document literacy, numeracy and problem solving [3]. The latest available data show that 41 % of Australians aged 15–74 had a level of health literacy that was adequate or above [4]. Health literacy (HL) deficits affect half of the overall American patient population, especially the elderly [5]. Low HL can make it difficult for patients to function effectively in the health care system [6]. Low health literacy has been consistently associated with poor health outcomes, including poorer health status [7–9], lack of knowledge about medical conditions and related care [10], lack of engagement with health care providers [11], decreased comprehension of medical information [10], mortality [12], and poorer use of preventive health services [10, 12], poorer self-reported health [10], and increased hospitalizations [10, 12] and higher health care costs [13, 14].

Health-related quality of life (HRQoL) refers to how individuals subjectively assess their own well-being and their ability to perform physical, psychological, and social functions [15]. Although there are many studies examining the relationship between HRQoL and heath literacy (HL) among patients with chronic diseases [10, 16], less is known about this relationship among patients without vascular disease or diabetes or who only have risk factors for these conditions.

The SF-36 and SF-12 are widely used measures of HRQoL. Investigators from numerous countries representing diverse cultures have determined that both measures are sensitive to differences in a number of socio-demographic variables, including gender [17, 18], age [17, 19], income [18, 20, 21], employment [18–20], education [20, 21] and country of birth [19]. In addition to patient demographic variables, patient lifestyle risk factors have been found to be associated with HRQoL. Other than low HL [7, 10], several studies found that smoking [22], poor diet [23, 24], alcohol risk [25], insufficient physical activity [26–28] and overweight [29–31] were negatively associated with HRQoL.

In this study, we investigated the relationship between HL and HRQoL in a large sample of adults without vascular disease or diabetes from four Australian states, using SF-12 version 2 after adjustment for both patient demographic and lifestyle risk factors. We are unaware of previous studies investigating the association between HL and HRQoL after simultaneous adjustment for both patient demographic and lifestyle risk factors in adults without vascular disease or diabetes. Based on the findings and some identified gaps in the literature, the following research questions were posed:

1. What are the differences in HL according to patients’ gender, age, place of birth, socio-economic status (home ownership, education and employment) and patient lifestyle risk factors (smoking, diet, alcohol, physical activity and BMI)?
2. What is the magnitude of the association between HL and HRQoL after adjustment for patient gender, age, place of birth, socio-economic status and lifestyle risk factors and are there any clinically significant differences in HRQoL among patient subgroups?
3. Are there any clinically significant differences in HRQoL between levels of each categorical independent variable within low or high HL patients?

## Methods

### Participants

The Preventive Evidence into Practice (PEP) study was a cluster randomised trial involving 30 practices in New South Wales (NSW), Victoria, South Australia and Queensland conducted in 2012 and 2013. This study aimed to examine uptake of guidelines for preventive care of vascular disease and diabetes in general practice [32]. A random sample of 160 patients (without known vascular diseases: cardiac disease, stroke, kidney disease or diabetes) aged between 40–69 years were invited by mail to participate in the study from each of the 30 general practices (4800 patients in total). Patients diagnosed and recorded in the medical record with these conditions or with psychosis, cognitive impairment (including dementia) or diagnosed substance abuse problems were excluded. We collected patient gender, age, place of birth, socio-economic status (home ownership, education and employment), and patients’ self-reported information on their risk factors (HL, weight, height, diet, smoking, alcohol status and physical activity) and HRQoL via mailed questionnaires.

Of the sample, 739 (15 %) patients provided consent and completed the baseline survey questionnaires. We compared demographic data of the participants in our survey with those from clinical audit data of 21,848 patients aged 40–69 from 27 of the 30 practices. Of these clinical audit patients, 61 % were females and their mean age was 52.9 years. The patients participating in the survey were more likely to be older and female compared to patients from the clinical audit. In this paper, a cross-sectional analysis of baseline data from the PEP cluster randomised trial was conducted.

A priori sample size calculation on the SF-12 mental component score estimated that, after adjustment for clustering, an average sample size of 25 patients from each of the 30 practices would have sufficient power (1-β = 0.8 and α = 0.05) to detect an effect size of 0.17 between low and high health literacy patients (previous studies on SF-12 indicated a cluster effect (ICC = intra-cluster correlation) of 0.011 for the MCS-12 [19]). The power is enough to detect even a small effect size. We computed Cohen’s d effect sizes. Cohen defined an effect size of 0.20 as small, 0.50 as moderate, and 0.80 or greater as large [33]. An effect size of more than 0.5 or half a standard deviation is considered to be clinically significant [17].

### Ethics

The study was approved by the National Research and Evaluation Ethics Committee of the Royal Australian College of General Practitioners (NREEC 10-002), and ratified by the Institutional Ethics Committees of participating universities. We obtained full informed written consent from participants.

### Instruments

The standard SF-12 version 2 is a 12-item questionnaire measuring physical and mental health [21, 34]. Use of the SF-12 version 2 has been recommended in preference to the original version 1 form for all new studies [35]. It is an abbreviated form of the SF-36 Health Survey, which is one of the most widely used instruments for assessing HRQoL [21]. Both instruments produce two summary scores – the Physical Component Summary (PCS) and the Mental Health Component Summary (MCS). These instruments have been validated for use in the USA, UK and many other European countries for large-scale health measurement and monitoring [21, 36]. For ease of interpretation, scores are standardized to population norms, with the mean score set at 50 (SD = 10); higher scores indicate better health. The SF-12 has been shown to have good validity and reliability [35] and sensitivity to change [37]. Previous research supports the use of the standard SF-12 in Australian settings, and it has been validated for Australia using standard US-derived scoring of the SF-12 summary scores [19, 22, 38, 39].

The same sample of patients completed the Health Literacy Management Scale (HeLMS) [40] along with the SF-12. The HeLMS has acceptable psychometric properties. It assesses a range of health literacy constructs important to patients when seeking, understanding and using health information within the healthcare system [40]. HeLMS is a patient completed instrument containing 5 point Likert scales assessing the level of ‘difficulty’ experienced with 29 items across 8 domains (with a range from 1: ‘unable to perform at all’, to 5 ‘experiencing no difficulty’). Five of the eight domains focus on individual abilities: patient’s attitude towards health, ability to understand health information, communication skills and pro-activeness, and skills in using health information. The remaining three domains focus on broader factors that influence these abilities: patient’s level of social support, socioeconomic status, and access to general practice healthcare. A mean score of < 4 in any of the eight average domain scores of HeLMS were considered as having insufficient or low health literacy. The HeLMS was chosen for this study as it has high quality assessment and high psychometric assessment [41]. The HeLMS is an Australian based tool. It also utilises a framework informed by interviews with patients from primary care and other healthcare settings [40, 42].

### Risk factors

We collected patient reported information via a mailed questionnaire. The questionnaire included demographic information and self-reported information relating to smoking status, nutritional and alcohol intake, physical activity, weight and height. The patient risk factors were low health literacy, smoking, diet, alcohol, physical activity and BMI. Any current smoking was considered a risk; poor diet was defined as eating ≤ 6 serves of fruits and vegetables daily and alcohol risk was defined as drinking more than two standard drinks in a typical day [43]. Physical activity scores were calculated using the frequency of vigorous and moderate physical activity per week (scored from 0 to 8). A score of less than 4 was considered inadequate physical activity in accordance with Australian guidelines [44]. Overweight was defined as having a Body Mass Index (BMI) of ≥25 [45]. BMI was calculated from patient reported weight and height.

### Other patient and practice variables

The independent variables included in the analyses were dichotomous patient reported risk factors and patient socio-demographic and practice characteristics. More details are shown in Table 1. The demographics of patients included as covariates in the analysis were patient reported gender, age, place of birth, home ownership, education and employment. Home and car ownership can be considered markers of economic status [46]. The practice characteristics included were the number of general practitioners (GPs) and number of nurses in a practice reported by the practice manager (Table 1).

**Table 1:** Distribution of health literacy according to patient and practice characteristics for 726 patients

Variable (definition)	Health literacy				
	Low (*n* = 351)		High (*n* = 375)		*p*-value*
	Number	%	Number	%	
Characteristics of patients					
Gender (*n* = 726)					
Male	115	32.8	106	28.3	0.188
Female	236	67.2	269	71.7	
Age, years (*n* = 725)					
40–54	177	50.4	141	37.7	0.001
55–70	174	49.6	233	62.3	
Place of birth (*n* = 726)					
Australia	261	74.4	283	75.7	0.731
Outside Australia	90	25.6	92	24.5	
Home ownership (*n* = 726)					
Owner occupied	308	87.7	347	92.5	0.030
Rented	43	12.3	28	7.5	
Education (*n* = 717)					
Degree/Diploma	187	54.0	179	48.2	0.121
Elementary/High school	159	46.0	192	51.8	
Employment (*n* = 719)					
Employed	241	69.7	234	62.7	0.003
Retired	50	14.5	91	24.4	
Unemployed^a^	55	15.9	48	12.9	
Patient risk factors					
Smoker (*n* = 721)					
Yes	40	11.5	21	5.6	0.005
No	309	88.5	351	94.4	
Poor diet (*n* = 726)					
Yes	292	83.2	293	78.1	0.085
No	59	16.8	82	21.9	
Alcohol risk (*n* = 721)					
Yes	24	6.9	16	4.3	0.131
No	325	93.1	356	95.7	
Insufficient physical activity (*n* = 715)					
Yes	217	62.7	175	47.4	<0.001
No	129	37.3	194	52.6	
Overweight (*n* = 696)					
Yes	224	67.5	188	51.6	<0.001
No	108	32.5	176	48.4	
Practice characteristics					
Practice size (*n* = 726)					
Small practice (1–3 GPs) (N* = 10)	129	36.8	119	31.7	0.154
Large practice (>3 GPs) (N* = 20)	222	63.2	256	68.3	
Number of nurses (*n* = 726)					
0–1 (N* = 14)	184	52.4	181	48.3	0.263
>1 (N* = 16)	167	47.6	194	51.7	

*p-values are for comparison of categories of each variable by health literacy using Pearson’s chi-square test

^a^Includes patients looking for work, studying full-time, looking after family and unable to work due to sickness or disability

N* = number of practices

### Statistical analyses

Summary physical (PCS-12) and mental (MCS-12) component scores were constructed using the standard SF-12 version 2 US algorithm which is empirically derived from the data of a US general population survey [35]. The dimensions as documented by Kontodimopoulos et al. [47] and Ware et al. [35] were confirmed and validated for Australia using standard US-derived scoring of the SF-12 summary scores [19].

Preliminary analyses were carried out using IBM SPSS version 20 (Tables 1 and 2). First, we examined research question 1: the association between the independent variables (all categorical) and health literacy (low or high HL) using the Pearson chi-squared test (Table 1). Then we examined research question 2: the magnitude of the association between HL and HRQoL. To address this initially, mean scores of PCS-12 or MCS-12 between low and high HL in each category of the independent variables were compared using independent t-tests (Table 2). Then the analyses for PCS-12 and MCS-12 were conducted to examine the association between HL and HRQoL after adjustment for categorical covariates using multilevel models (Tables 3 and 4). First, we fitted a baseline variance component or empty model (no independent variables) for each component scores followed by the model with HL only and then the main model. The main model expands the model with HL only by including other patient and practice variables including risk factors (Tables 3 and 4). Then we added interactions between HL and potential independent variables to the main model. Parameter estimates of fixed effects were tested for significance using a *t*-test, determined by dividing the estimated coefficients by their standard errors. The significance of the random variance estimates was assessed using the Wald joint chi-squared test [48]. The Deviance Statistic was used to test whether additional model predictors improved the fit of the model [48, 49]. Finally, we examined research question 3: clinically significant differences in HRQoL within low or high HL patients. The comparison of mean scores of PCS-12 and MCS-12 for categories of independent variables within low HL or high HL was carried out with independent t-tests or one-way analysis of variance (Table 2). Then we carried out analyses for PCS-12 or MCS-12 stratified by HL (separate model for low or high HL) with adjustment for confounding factors using multilevel regression models (Table 5). This model also helps to interpret the interactions shown in Tables 3 and 4.

**Table 2:** Unadjusted mean and standard deviation of PCS-12 and MCS-12 scores by health literacy (HL) status

Variable (definition)	PCS-12			MCS-12		
	Low HL	High HL		Low HL	High HL HL	
	Mean (SD)	Mean (SD)	*p*-value^a^	Mean (SD)	Mean (SD)	*p*-value^a^
Overall score	46.2 (12.2)	51.9 (7.9)	<0.001	47.3 (11.0)	54.4 (6.9)	<0.001
Characteristics of patients						
Gender						
Male	48.0 (11.0)*	52.6 (7.5)	0.001	47.1 (10.5)	55.4 (6.3)	<0.001
Female	45.3 (12.6)	51.6 (8.0)	<0.001	47.4 (11.3)	53.9 (7.1)	<0.001
Age, years						
40–54	47.6 (11.6)*	52.6 (7.6)	<0.001	45.9 (11.0)	53.3 (7.3)	<0.001
55–70	44.7 (12.6)	51.5 (8.0)	<0.001	48.8 (10.8)*	55.0 (6.6)*	<0.001
Place of birth						
Australia	45.9 (12.6)	51.9 (8.1)	<0.001	47.1 (11.3)	54.2 (6.8)	<0.001
Outside Australia	47.1 (10.6)	52.0 (7.3)	0.001	47.9 (10.2)	54.8 (7.4)	<0.001
Home ownership						
Owner occupied	46.4 (11.8)	51.9 (7.9)	<0.001	47.7 (10.7)	54.4 (6.8)	<0.001
Rented	44.3 (14.3)	51.9 (8.4)	0.011	44.5 (13.2)	54.2 (8.4)	0.001
Education						
Degree/Diploma	48.9 (10.6)***	53.1 (7.3)**	<0.001	48.0 (10.1)	53.9 (7.1)	<0.001
Elementary/High school	43.0 (13.0)	50.7 (8.3)	<0.001	46.2 (12.0)	54.8 (6.8)	<0.001
Employment						
Employed	48.7 (10.4)*	53.3 (6.8)*	<0.001	47.7 (10.5)*	53.7 (6.9)	<0.001
Retired	43.0 (13.7)*	49.9 (8.7)	0.004	49.3 (11.5)*	56.1 (6.5)	0.001
Unemployed^b^	38.5 (13.9)	49.2 (10.0)	<0.001	43.7 (12.6)	54.3 (7.4)	<0.001
Patient risk factors						
Smoker						
Yes	41.6 (13.3)	52.8 ((10.2)	0.001	45.2 (12.1)	53.4(8.7)	0.012
No	46.7(12.0)*	51.9(7.7)	<0.001	47.5(10.9)	54.4(6.8)	<0.001
Poor diet						
Yes	46.3 (11.8)	51.8 (8.0)	<0.001	47.1 (11.0)	54.2 (6.9)	<0.001
No	45.4 (13.8)	52.4 (7.6)	0.001	48.3 (11.3)	54.9 (7.0)	<0.001
Alcohol risk						
Yes	45.7 (12.4)	54.0 (5.5)	0.008	46.7 (12.9)	53.0 (8.2)	0.115
No	46.2 (12.2)	51.8 (8.0)	<0.001	47.3 (10.9)	54.4 (6.9)	<0.001
Insufficient physical activity						
Yes	44.4 (12.9)	50.4 (8.8)	<0.001	45.4 (11.7)	54.1 (7.1)	<0.001
No	49.2 (10.1)**	53.4 (6.7)***	<0.001	50.5 (9.1)***	54.4 (6.7)	<0.001
Overweight						
Yes	44.8 (12.4)	50.6 (8.3)	<0.001	47.4 (11.5)	55.0 (6.9)	<0.001
No	48.3 (11.8)*	53.3 (7.4)*	<0.001	47.5 (10.5)	53.8 (6.9)	<0.001
Characteristics of practice						
small practice (1–3 GPs)	45.2 (11.7)	51.9 (7.5)	<0.001	46.7 (11.9)	54.2 (7.2)	<0.001
large practice (>3 GPs)	46.8 (12.4)	51.9 (8.1)	<0.001	47.7 (10.5)	54.4 (6.8)	<0.001
Number of nurses						
0 –1	47.2 (11.5)	52.6 (6.9)	<0.001	47.3 (10.9)	54.4 (6.4)	<0.001
>1	45.0 (12.9)	51.4 (8.5)	<0.001	47.3 (11.2)	54.4 (7.4)	<0.001

*P <0.05, **P <0.01, and ***P <0.001 are for comparison of within low or high HL differences between score of a category with the category of the lowest score using independent t-test or one-way analysis of variance

^a^ *p*-values are for comparison of difference of each category of patient and practice characteristics using independent t- test

^b^Includes patients looking for work, studying full-time, looking after family and unable to work due to sickness or disability

Number in each category is as shown in Table 1 subject to small number of missing values in SF12 items

**Table 3:** Estimates of regression coefficient of multilevel regression analysis for practice and patient characteristics for physical components score (PCS-12)

Parameters (reference category)	Regression coefficients (standard error)			
	Empty model	Model only with health literacy	Main model with all patient and practice variables	Model with interactions
Patient demographics				
Intercept	48.78	51.66	52.90	55.49
Female patients (male)			−0.72 (0.90)	−0.58 (0.88)
Age, years				
55–70 (40–54)			−0.92 (0.88)	0.87 (1.22)
Born in Australia (born in outside Australia)			−0.93 (0.90)	−1.11 (0.89)
Owner-occupier (rented)			−0.46 (1.39)	−0.95 (1.37)
College/university (elementary/high School)			2.06 (0.83)*	2.83 (1.53)
Employed patients (unemployed)			6.02 (1.20)***	2.62 (1.71)
Retired patients (unemployed)			3.15 (1.40)*	−0.17 (1.86)
Patient risk factors				
Insufficient health literacy (sufficient health literacy)		−5.57 (0.77)	−5.35 (0.79)***	−9.55 (2.47)***
Smoker (non-smoker)			−2.85 (1.52)	0.75 (2.32)
Poor diet (having adequate fruits and vegetables intake)			−0.60 (0.96)	−0.33 (0.94)
Alcohol risk (drinking ≤2 standards drinks in a day)			1.90 (1.69)	2.89 (1.67)
Insufficient physical activity (adequate physical activity)			−3.07 (0.78)***	−3.15 (0.76)***
Overweight (having normal range BMI)			−1.84 (0.81)*	−1.96 (0.80)*
Patient interaction effects				
Low health literacy × 55–70				−3.82 (1.70)*
Low health literacy × degree				2.83 (1.53)^a^*
Low health literacy × employed				6.13 (2.30)**
Low health literacy × retired				6.16 (2.74)*
Low health literacy × smokers				−6.42 (3.02)*
Practice characteristics				
Size 1–3 general practitioners (4 or more GPs)			−1.42 (1.13)	−1.38 (1.14)
More than one nurses (one or no nurse)			−1.65 (1.09)	−1.65 (2.74)
Random effects				
Variance between general practices (standard errors)	6.28 (2.93)	5.31 (2.58)	1.77 (1.51)	2.02 (1.54)
% variance explained^b^		15.5	71.8	67.8
Variance between patients (standard errors)	107.45 (5.90)***	97.65 (5.41)***	85.70 (4.97)***	81.87 (4.75)***
% variance explained^b^		9.1	20.2	23.7

*P <0.05, **P <0.01, ***P <0.001

^a^Borderlind significant (*P* = 0.06)

^b^Explained variance using the variance in the empty model as reference

**Table 4:** Estimates of regression coefficient of multilevel regression analysis for practice and patient characteristics for mental components score (MCS-12)

Parameters (reference category)	Regression coefficients (standard error)			
	Empty Model	Model only with health literacy	Main model with all patient and practice variables	Model with interactions
Patient demographics				
Intercept	50.97	54.36	52.66	54.76
Female patients (male)			0.19 (0.84)	0.19 (0.83)
Age, years				
55–70 (40–54)			2.13 (0.83)*	0.73 (1.15)
Born in Australia (born in outside Australia)			−0.61 (0.85)	−0.77 (0.84)
Owner-occupier (rented)			0.23 (1.31)	–0.18 (1.30)
College/university (elementary/high School)			0.01 (0.77)	0.08 (0.77)
Employed patients (unemployed)			2.42 (1.13)*	0.17 (1.62)
Retired patients (unemployed)			2.87 (1.32)*	1.92 (1.76)
Patient risk factors				
Insufficient health literacy (sufficient health literacy)		–7.02 (0.70)	–6.43 (0.74)***	–8.38 (2.43)***
Smoker (non-smoker)			–1.33 (1.44)	–1.17 (1.43)
Poor diet (having adequate fruits and vegetables intake)			–0.34 (0.91)	–0.20 (0.90)
Alcohol risk (drinking ≤2 standards drinks in a day)			0.07 (1.59)	0.27 (1.58)
Insufficient physical activity (adequate physical activity)			–2.56 (0.73)***	–0.56 (0.97)
Overweight (having normal range BMI)			0.78 (0.76)	0.81 (0.76)
Patient interaction effects				
Low health literacy × 55–70				2.70 (1.51)^a*^
Low health literacy × employed				3.95 (2.01)*
Low health literacy × retired				1.03 (2.60)
Low health literacy × Insufficient physical activity				−4.42 (1.44)**
Practice characteristics				
Size 1–3 general practitioners (4 or more GPs)			−0.88 (0.87)	−0.06 (0.91)
More than one nurses (one or no nurse)			−0.62 (0.85)	−0.85 (0.88)
Random effects				
Variance between general practices (standard errors)	1.50 (1.44)	0.25 (0.94)	0.00 (0.00)	0.29 (0.94)
% variance explained^b^		83.5	100.0	80.7
Variance between patients (standard errors)	95.24 (5.22)***	82.64 (4.57)***	77.39 (4.40)***	75.16 (4.36)***
% variance explained^b^		13.2	18.7	21.1

*P <0.05, **P <0.01, ***P <0.001

^a^Borderlind significant (*P* = 0.07)

^b^Explained variance using the variance in the empty model as reference

**Table 5:** Estimates of regression coefficient of multilevel regression analysis for practice and patient characteristics stratified by low and high health literacy (HL) for physical and mental components score

Parameters (reference category)	Regression coefficients (standard error)			
	Physical components score		Mental components score	
	Low HL	High HL	Low HL	High HL
Patient demographics				
Intercept	49.17	52.60	45.11	55.76
Female patients (male)	−0.49 (1.48)	−0.99 (0.98)	1.38 (1.43)	−0.93 (0.91)
Age, years				
55–70 (40–54)	−2.89 (1.42)*	0.93 (0.99)	3.56 (1.39)*	0.55 (0.91)
Born in Australia (born in outside Australia)	−2.40 (1.48)	0.15 (1.0)	−1.53 (1.45)	−0.07 (0.91)
Owner-occupier (rented)	−1.37 (2.05)	−0.03 (1.76)	0.54 (2.01)	−0.75 (1.62)
College/university (elementary/high School)	2.90 (1.39)*	1.29 (0.89)	0.79 (1.35)	−0.73 (0.82)
Employed patients (unemployed)	8.70 (1.89)***	3.15 (1.40)*	4.17 (1.85)*	0.49 (1.28)
Retired patients (unemployed)	6.00 (2.41)*	−0.11 (1.51)	2.92 (2.36)	1.98 (1.39)
Patient risk factors				
Smoker (non-smoker)	−6.43 (2.30)**	0.85 (1.91)	−1.89 (2.25)	−0.45 (1.75)
Poor diet (having adequate fruits and vegetables intake)	0.67 (1.70)	−1.06 (0.99)	0.49 (1.67)	−0.73 (0.91)
Alcohol risk (drinking ≤2 standards drinks in a day)	4.51 (2.60)	1.63 (2.05)	2.26 (2.54)	−1.38 (1.88)
Insufficient physical activity (adequate physical activity)	−4.02 (1.32)**	−2.41 (0.83)**	−5.14 (1.29)***	−0.53 (0.76)
Overweight (having normal range BMI)	−2.86 (1.41)*	−1.68 (0.85)*	1.17 (1.38)	0.53 (0.78)
Practice characteristics				
Size 1–3 general practitioners (4 or more GPs)	−2.69 (2.13)	−0.24 (1.03)	−2.16 (1.71)	0.02 (0.94)
More than one nurses (one or no nurse)	−3.49 (2.07)	−0.59 (0.97)	−1.68 (1.69)	−0.25 (0.89)
Random effects				
Variance between general practices (standard errors)	9.14 (5.41)	0.00 (0.00)	2.79 (3.43)	0.00 (0.00)
Variance between patients (standard errors)	107.43 (9.35)***	53.60 (4.18)***	105.15 (9.12)***	45.24 (3.53)***

*P <0.05, **P <0.01, ***P < 0.001

Multilevel analysis used MLwiN 2.30 [48] adjusted for clustering of patients (level 1) within general practice (level 2) [18, 19]. Two-sided p-values of less than 0.05 were considered statistically significant.

## Results

The mean age was 55.5 years (interquartile range (IQR) = 49–62) and 69 % were females.

The low and high HL comparison for demographic characteristics and risk factors of patients and practice characteristics is presented in Table 1. The HL score was available for more than 98 % of patients. Low HL patients tended to be younger (40–54 years) than high HL patients (50 % vs 38 %, *P* = 0.001). High HL patients tended to be retired (24 % vs 14 %, *P* = 0.003) and 5 % more owner-occupiers (*P* = 0.030) than low HL patients. Low HL patients were more likely to be smokers (12 % vs 6 %, *P* = 0.005), to have insufficient physical activity (63 % vs 47 %, *P* < 0.001) and be overweight (68 % vs 52 %, *P* < 0.001). The distribution of practice characteristics over the low and high HL groups was similar (Table 1).

The overall means of PCS-12 scores for low and high HL were 46.2 (SD = 12.2) and 51.9 (SD = 7.9) respectively (Table 2). Similarly, overall mean MCS-12 scores were 47.3 (SD = 11.0) for low HL and 54.4 (SD = 6.9) for high HL. The two summary scores were available for 94 % of patients. Table 2 shows the differences between PCS-12 or MCS-12 scores of low and high HL patients for the subcategories of patient and practice characteristics and patient risk factors (Table 2). Low HL patients reported poorer physical health than high HL for all categories of independent variables. Similarly, low HL patients reported poorer mental health than high HL for all categories of independent variables except for alcohol risk group (Table 2). Among low HL patients, those who were female, older, not well educated, unemployed, a smoker, overweight or had insufficient physical activity were likely to have lower physical health. The differences in physical health between less well educated and well educated (43.0 vs. 48.9, effect size = 0.50) and unemployed and employed (38.5 vs 48.7, effect size = 0.92) were the only clinically significant differences for low HL patients (Table 2). The education and employment remained significant in predicting physical health after adjustment for confounding effects with multilevel analysis for low HL patients (Table 5). Similarly, among high HL patients those who were less well educated, unemployed, retired, overweight or had insufficient physical activity were likely to have lower physical health. Only the difference in physical health between unemployed and employed (49.2 vs. 53.3, effect size = 0.55) was clinically significant for high HL patients (Table 2). The employment remained significant in predicting physical health after adjustment for confounding effects for high HL patients (Table 5). Among low HL patients, those who were younger, unemployed, and had insufficient physical activity tended to have lower mental health (Table 5). Similarly among high HL patients those who were younger tended to have lower mental health (Table 2). All the differences in mental health were not clinically significant. The above effects were not adjusted for confounding effects.

The associations between low and high health literacy and PCS-12 and MCS-12 are presented in the multivariate multilevel regression analyses in Tables 3 and 4 and separately for patients with high and low health literacy in Table 5.

Patients with low health literacy were likely to have lower physical health with a large clinically significant effect size of ≥ 0.56 (B (regression coefficient) ≤ - 5.4, *P* < 0.001) and lower mental health with a large clinically significant effect size of ≥ 0.78 (B ≤ −6.4, *P* < 0.001) compared to those with higher HL after adjustment for confounding factors (Tables 3 and 4). After accounting for confounding factors, regression coefficients for lower HL in the main model were only marginally increased for PCS-12 (from −5.57 to −5.35) and for MCS-12 (from −7.02 to −6.43). This shows that other patient factors had negligible influence on the strong association between HL and PCS-12 or HL and MCS-12. MCS-12 scores increased with age (*P* < 0.05). Gender or home ownership were not significantly associated with either component score. Patients who were employed (effect size = 0.75, B = 6.0, *P* < 0.001) or retired (effect size = 0.35, B = 3.2, *P* < 0.05) were likely to have higher PCS-12 scores. Similarly, patients who were employed (effect size = 0.23, B = 2.4, *P* < 0.05) or retired (effect size = 0.55, B = 2.9, *P* < 0.05) tended to have higher MCS-12 scores than unemployed. Employment interacted with HL for PCS-12 and MCS-12 (Tables 3 and 4). Education was positively associated with PCS-12 (effect size = 0.34, *P* < 0.05). Older patients (55–69 years) tended to have higher MCS-12 scores than younger patients (40–54, years) (effect size = 0.33, *P* < 0.05). However retirement and age were no longer significant in the separate analysis of high HL patients (Table 5).

Patients with insufficient physical activity were more likely to have a lower physical health (effect size = 0.42, B = -3.1, *P* < 0.001) and lower mental health (effect size = 0.37, B = −2.6, *P* < 0.001) than those with sufficient physical activity. Being overweight had a negative effect on PCS-12 (effect size = 0.41, B = −1.8, *P* < 0.05). Poor diet or alcohol risk were not associated with PCS-12 scores or MCS-12 scores (Tables 3 and 4). There was an interaction between smokers and HL for PCS-12 and physical activity and HL for MCS_12. Smokers had a negative effect on physical health (Tables 3 and 5) and physical activity had a negative effect on mental health (Tables 4 and 5) for low HL patients. Practice characteristics were also not associated with either PCS-12 or MCS-12 scores.

### Percentage of variance explained

At the practice level (level 2), 72 % (of which HL explained 16 %) and 100 % (of which HL explained 84 %) of the practice variances in PCS-12 and MCS-12 were explained respectively by the variables used in the main model (Tables 3 and 4). At the patient level (level 1) the variance in PCS-12 explained was 20 % of which 9 % was explained by HL and the remaining 11 % was due to all other independent variables (Table 3). Similarly, at the patient level (level 1) the variance in MCS-12 scores explained was 19 % of which 13 % was explained by HL and the remaining 6 % was due to all other independent variables (Table 4). Remarkably, HL accounted for 45 and 70 % of the total between patient variance explained in PCS-12 and MCS-12 respectively.

## Discussion

To the best of our knowledge, this is the first study to investigate the impact of HL on HRQoL after simultaneous adjustment for both patient demographics and lifestyle risk factors in adults without chronic vascular disease or diabetes. We found two studies which examined the effect of HL on PCS-12 and MCS-12 after adjustment for confounding factors in patients with chronic conditions. The first study [50] comprised of 3260 elderly (≥65) patients with ten chronic health conditions (Heart attack, Stroke, Asthma, Cancer, Diabetes etc.). The effects of inadequate HL (Reference: adequate) on PCS-12 (B = −2.53, *P* < 0.001) and MCS-12 (B = 1.41, *P* < 0.001) were weaker than those of our study. The second study [51] included 1581 men with newly diagnosed clinically localized prostate cancer from a population based study. In this study, patients with higher health literacy had better MCS-12 (B = 2, *P* < 0.04) than low HL. However, the association between HL and PCS-12 was significant only in the bivariate model. Again the difference of HRQoL between low and high HL patients was weaker than those of our study. This may be due to the lower HRQoL of chronically ill patients and that they have less variation when compared to better HRQoL of patients without vascular disease or diabetes. For example, the overall average of PCS-12 in the current study was 49.0 compared to 42.4 in Australian patients with diabetes or cardiovascular disease [19].

This study provides comprehensive data on the association between the HL and self-rated physical and mental health of Australian adults without previously diagnosed vascular disease or diabetes. Our findings demonstrated that lower HL patients reported clinically significant poorer physical health and mental health than higher HL patients with HL accounting for 45 and 70 % of the total between patient variance explained in PCS-12 and MCS-12 respectively. A potential explanation of these negative associations between low health literacy and physical and mental domains of HRQoL may be that low health literacy can make it difficult for patients to effectively navigate the health care system [6]. Patients with lower HL may have difficulties with complex health tasks and ability to seek and understand health information [40], have limited access to the health care [40], lack engagement with health care providers [11], and have poorer uptake of preventive health care [10, 12]. Patients with lower health literacy tend to have difficulties with communication, which prevent them from asking questions, clearly expressing their concerns, emotions, and needs to providers and seeking additional services such as support for mental health [10, 52]. Being able to recognise low health literacy is important in general practice as there is good [53] or mixed [54] evidence that tailoring health related communication to those with low health literacy can improve health outcomes.

Few studies have examined the impact of HL and its interaction with socio-demographics factors on HRQoL. For PCS-12, our study showed some significant interaction effects between HL and age, HL and education (borderline with *P* = 0.06), HL and employed or retired, and HL and smokers (Table 3). Similarly for MCS-12, interaction effects between HL and employment, HL and age (borderline), HL and physical activity were significant (Table 4). These interaction effects for PCS-12 and MCS-12 were explored in Table 5. Among lower HL patients, educational attainment was positively associated with physical heath. For high HL patients, this association between education and physical health was not significant after adjustment for confounding factors (Table 5). This suggests that the combination of lower HL and lower educational attainment is particularly important in physical health. Practitioners need to be alert to problems with communication and adherence to treatment plans and access to services as these patients encounter double disadvantage. Education had no association with the mental health of low or high HL patients (Tables 2, 4 and 5).

There are no other studies we are aware of that examined the impact of the association between health literacy and employment in predicting HRQoL. The interaction between HL and employment status showed that the negative impact of unemployment was greater in low HL patients than high HL. We found that the negative impact of low health literacy in unemployed patients with 8.7 lower PCS-12 than employed and 6.0 lower PCS -12 than retired (Tables 3 and 5). Similarly, unemployed patients had 4.2 lower MCS-12 than employed for low HL patients (Tables 4 and 5). Consistent with other research, lower socio-economic groups reported lower PCS-12 and MCS-12 [18, 20, 55].

The finding that mental health was higher in the older age group is consistent with our previous research [19]. The older age had a negative effect on PCS-12 and positive effect on MCS-12 for low HL patients.

We found that almost half the patients in this study met the criteria for insufficient health literacy. This is consistent with studies in primary care in other developed countries [56] and the prevalence reported in the Australian community [57]. Patients with low HL were more likely to be smokers, report being overweight or obese, and exercise inadequately. In multivariate analysis, inadequate physical activity tended to have a negative effect on physical health (Table 5). Being overweight also had a negative effect on physical health (Table 5). This supports findings from previous studies demonstrating associations between HRQoL and physical activity [31, 58] or BMI [31, 59]. The analyses showed that life style risk factors interacted with HL (Tables 3 and 4). Low HL smokers were likely to have 6.4 lower PCS-12 than non-smokers and low HL patients with insufficient physical activity tended to have 5.1 lower MCS-12 after adjustment for confounding factors (Tables 3, 4 and 5). Our results extend findings from previous studies by demonstrating the possible beneficial association of regular physical activity, non-smoking or normal weight with HRQoL is more relevant to low HL patients.

There are a number of limitations to this study. Patients identified by the practice as being unable to read English, with psychosis, cognitive impairment, diagnosed substance abuse problems or severe mental illness were excluded from the study. Others may have self excluded in their response to the written invitation to participate, particularly patients with low health literacy who may not have understood why it was important to be involved, or what might have been required of them. It is possible that non-respondents might have assessed their physical and mental health differently from those who responded and had lower health literacy. The response rate was also low (15 % compared to 30 % in our previous study [60], possibly due to the recruitment method or because patients no longer wished to attend the practice). The patients responding to the survey were older and more were female compared to patients from the clinical audit in the same practices. Participants of this study were predominantly not from low socio-economic groups. The majority of them were Australian-born, had a degree or diploma, were in paid employment and owned their home. It is possible that due to selection bias more high health literacy patients responded and this could have diluted the effects of this study.

The strengths of the study include a broad sample of 739 patients from 30 general practices in four states of Australia, the adjustment for confounding for both patient (demographic and lifestyle risk factors) and practice variables and the correction for practice level clustering with multilevel modelling.

## Conclusions

Addressing health literacy related barriers may help reduce disparities in HRQoL. Being able to recognise low health literacy is important in general practice. Tailoring health related communication and support for appropriate health services use for those with lower health literacy may improve health outcomes, including HRQoL. Strategies to improve health outcomes and quality of life need to address both system demands and community capacity. The former includes tailoring health related communication to the needs of those with low health literacy and providing support for their appropriate health services use. The latter includes more effective education and information to enhance patient and community health literacy. The role of general practice may also be to support patients to improve their health literacy so that they are better able to access and navigate preventive care. It seems to be important that the health service environment be sensitive to the needs of patients with low health literacy. We observed that lower level of education or unemployment was associated with worse physical health for low HL patients in our cross-sectional study. Actions need to be taken to minimize the disadvantage suffered by low health literacy patients, particularly with lower level of education and unemployed. Further research is needed to clarify the impact of the combination of low HL and lower level of education or unemployment on HRQoL.

### Availability of data

The data set used to generate the results of this paper contains identified patient data. For reasons of privacy we have therefore provided the data on which these results are based within the tables provided. The full data set will be made available for open access after the completion of the project activities. This will be made available from the UNSWorks and the records are published on online portal Research Data Australia (RDA).

## Abbreviations

B: Regression coefficient
BMI: Body Mass Index
HeLMS: Health Literacy Management Scale
HL: Health literacy
HRQOL: Health-related quality of life
ICC: Intra-cluster correlation
MCS-12: Mental Component Score derived from the SF-12
NSW: New South Wales
PCS-12: Physical Component Score derived from the SF-12
PEP: Preventive Evidence into Practice
RACGP: Royal Australian College of General Practitioners
SD: Standard deviation
SF-12: Short Form 12-item Health Survey
SF-36: Short Form 36-item Health Survey
US: United States
